# Biosecurity Assessments for Emerging Transdisciplinary Biotechnologies: Revisiting Biodefense in an Age of Synthetic Biology

**DOI:** 10.1089/apb.2024.0005

**Published:** 2024-09-18

**Authors:** Diane DiEuliis, Michael J. Imperiale, Kavita M. Berger

**Affiliations:** ^1^ Center for the Study of Weapons of Mass Destruction, National Defense University, Washington, DC, USA.; ^2^ University of Michigan, Ann Arbor, Michigan, USA.; ^3^ Board on Life Sciences, National Academies of Sciences, Engineering, and Medicine, Washington, DC, USA.

**Keywords:** engineering biology, synthetic biology, generative artificial intelligence, biological security, biohybrid, microbiome

## Abstract

**Introduction:**

Rapid advances in biotechnologies and transdisciplinary research are enhancing the ability to perform full-scale engineering of biology, contributing to worldwide efforts to create bioengineered plants, medicines, and commodities, which promise sustainability and innovative properties.

**Objective:**

This rapidly evolving biotechnology landscape is prompting focused scrutiny on biosecurity frameworks in place to mitigate harmful exploitation of biotechnology by state and non-state actors. Concerns about biosafety and biosecurity of engineering biology research have existed for decades as views about how advances in this and associated fields might provide new capabilities to malicious actors. This article considers biosecurity concerns using examples of research advances in engineering biology.

**Methods:**

The authors explore risk assessment and mitigation of transdisciplinary biotechnology research and development, using the framework developed in the National Academies' study on *Biodefense in an Age of Synthetic Biology*.

**Results:**

The Synthetic Biology Assessment Framework focuses on risks of using advanced approaches and technologies to enhance or create novel pathogens and toxins. The field of engineering biology continues to advance at a pace that challenges current risk assessment frameworks.

**Conclusions:**

This framework likely is sufficient to assess new science and technology advances affecting conventional biological agents. However, the risk assessment framework may have limited applicability for technologies that are not usable with conventional biological agents and result in economic or broader national security concerns. Finally, the vast majority of discourse has been focused primarily on risks rather than benefits, and analyzing both in future evaluations is critical to balancing scientific progress with risk reduction.

## Introduction

For the past few decades, biotechnology has seen steady iterative advances punctuated by more disruptive discoveries that rapidly accelerate the field. Along with this trajectory, policy and governance for biosafety and biosecurity have also evolved, although frequently in (often slow) reaction to such disruption rather than in parallel. For example, concern over the discovery of recombinant DNA in the late 1960s and early 1970s prompted the famous Asilomar Conference, resulting in the “NIH Guidelines” that still is relied upon today as the benchmark for biosafety.

Similarly, concern over unauthorized access to pathogens, non-state actor use of biological agents, and state development of biological agents as weapons established much of the foundation for current policy frameworks within the United States and internationally. The recreation of poliovirus in 2002,^1^ creation of the first synthetic cell,^2,3^ and the first use of CRISPR in humans^4,5^ are among the findings that have culminated in the current landscape of biosafety and biosecurity policies.^6^

## Landscape of Engineering Biology

Rapid advances in biotechnologies and transdisciplinary research are enhancing the ability to perform full-scale engineering of biology, contributing to worldwide efforts to create bioengineered plants, medicines, and commodities that promise sustainability and innovative properties.^7^ Engineering biology, which previously was referred to as synthetic biology, now can produce fabrics, drugs, and building materials,^8^ and microbiomes can be purposefully designed for therapeutic benefits (two microbiome treatments have been approved by the Food and Drug Administration [FDA] as of this writing).^9^

Furthermore, engineering biology researchers are seeking to develop biological alternatives to petroleum-based approaches for degrading plastic waste,^10,11^ mining metals from Earth and discarded electronics,^12–16^ addressing environmental pollution,^17^ and many other applications. These advances have been facilitated by various actors to develop high-throughput screens, apply machine learning and artificial intelligence (AI) to the design of new biological systems and molecules, and lower barriers to entry into the field both in knowledge and financial resources.

## Recent Biosecurity Policy Context

To harness advances in engineering biology, recent U.S. government initiatives have focused on biotechnological research, development, and infrastructure. The CHIPS and Science Act calls for a national genomic sequencing strategy; investments in a National Engineering Biology Research and Development initiative; the scale-up of biomanufacturing processes; and considerations of ethical, legal, safety, security, environmental, and other societal issues associated with engineering biology research and development.^18^

The *Executive Order on Advancing Biotechnology and Biomanufacturing Innovation for a Sustainable, Safe, and Secure American Bioeconomy* pushes for foundational genetic engineering technologies to “write circuitry and predictably program biology.”^19^ Finally, the *Bold Goals for U.S. Biotechnology and Biomanufacturing* prioritizes achievement of five short- and long-term goals for engineering biology research and development efforts, including addressing challenges in climate change, food and agriculture, supply chain resilience, human health, and infrastructure and workforce for bio-based solutions.^20^

These policies call for biosafety and biosecurity guardrails that accompany the bioeconomy advances, leaving the details for how to assess and address these risks to the policy implementation phase.

This rapidly evolving biotechnology landscape is prompting focused scrutiny on biosecurity frameworks in place to mitigate harmful exploitation of biotechnology by state and non-state actors. Concerns about biosafety and biosecurity of engineering biology research have existed for decades as views about how advances in this and associated fields might provide new capabilities to malicious actors. These concerns often focused on the methods, knowledge, and technologies that reduce access barriers to pathogens and toxins and enable creation of novel organisms having novel functions by such actors.

As these concerns grew, the National Academies of Sciences, Engineering, and Medicine conducted a study, *Biodefense in an Age of Synthetic Biology*,^21^ which produced a framework for assessing the risk of engineering biological capabilities, building on and expanding considerations first introduced in the 2004 National Academies' report, *Biotechnology in an Age of Terrorism*.^22^ The study on synthetic biology, which was published in 2018, focused on the “design-build-test” cycle of engineering biology and points within the cycle that could be impacted by emerging technological advances.

Although the framework focuses on assessing harms of engineering biology to humans, it had broader implications that go beyond that singular risk. Shortly thereafter, the National Academies published a study in 2020 on *Safeguarding the Bioeconomy*, which included a broader set of risks associated with the bioeconomy, specifically from innovations derived from engineering biology and associated fields and their scaling up to create new foods, fuels, medicines, and other commodities and services.^7^ This study expanded the risk profile beyond risks to human health, to include economic security and competitiveness, and introduced a now widely adopted “promote and protect” paradigm to address security risks to the bioeconomy.

These concepts were integrated into the Executive Order on biotechnology and biomanufacturing mentioned earlier. More recently, the intense focus on the use of AI, specifically large language models (LLMs), in engineering biology and other biotechnology fields, has led to numerous reports by nongovernmental and intergovernmental entities on addressing potential biosecurity risks of AI and the life sciences.^23–30^

In 2023, the White House issued *Executive Order on the Safe, Secure, and Trustworthy Development and Use of Artificial Intelligence*, which calls for addressing “AI systems' most pressing security risks – including with respect to biotechnology…,” highlighting concerns about AI “substantially lowering the barrier of entry for non-experts to design, synthesize, acquire, or use chemical, biological, radiological, or nuclear (CBRN) weapons,” and recognizing the risks and benefits of generative AI to biosecurity.^31^

This context of rapidly advancing policy development to simultaneously promote research and development in engineering biology and associated fields protects these advances from theft or otherwise unauthorized or unfair access, and prevents and reduces biosecurity risks that will perpetuate and complicate existing challenges with which scientists, security experts, and policy makers struggle.

This article explores one aspect of modern biosecurity policy issues, specifically risk assessment and mitigation of transdisciplinary biotechnology research and development, using the framework developed in the National Academies' study on *Biodefense in an Age of Synthetic Biology*. This and other National Academies' reports that are referenced in this article are the result of contributions by committee members, who generously volunteer their time and expertise to consider the critical issues relevant to each report.

This article builds on their efforts through the National Academies' report and is supplemented by published scientific literature. Furthermore, this article evaluates how specific key conclusions and recommendations from this report have held up in light of recent and anticipated advances in engineering biology and related fields.

## Risk Assessment Framework for Synthetic Biology from *Biodefense in an Age of Synthetic Biology*

In 2016, the Department of Defense (DoD), along with other U.S. government agencies involved in biodefense, asked the National Academies of Science, Engineering, and Medicine to conduct a study about the biodefense considerations of synthetic biology. The sponsors clearly recognized the tremendous potential benefits of the technologies, but also were concerned about the possibility for misuse. The study was conducted in two parts. The first was to devise a framework that could be used to assess potential vulnerabilities and the second was to apply the framework to the state of the field as it existed at that time, as well as provide a timeframe for when new vulnerabilities might arise.

The goal of the framework was to provide a basis for identifying and prioritizing potential areas of concern created by current and future capabilities in synthetic biology. A framework is a valuable tool for parsing the biotechnology landscape as it evolves; it facilitates the identification of bottlenecks and barriers and can be used to monitor advances in technology and knowledge that change what is possible. The framework created was intended not only for use by technical experts in synthetic biology and biotechnology, but also for experts in complementary areas (e.g., intelligence and public health).

**Figure 1.**
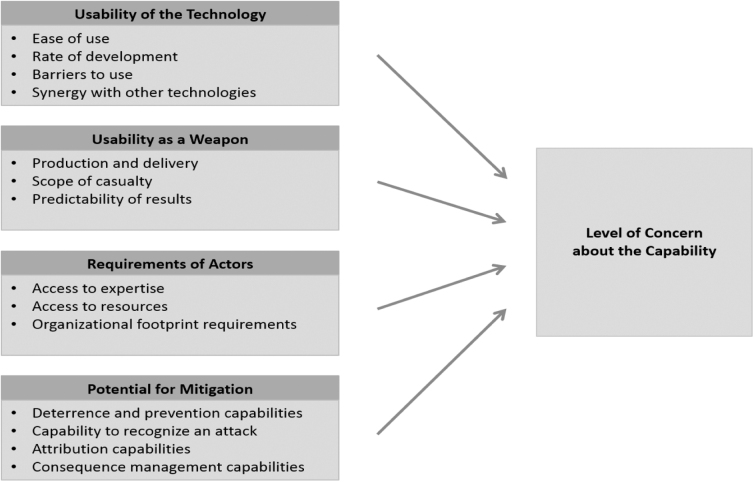
Framework for assessing concern. *Source:* National Academies of Sciences, Engineering, and Medicine.^21^

The report describes four factors that were built into the framework: usability of the technology, usability as a weapon, requirements of actors, and potential for mitigation. The details of the considerations to be taken into account for each factor are shown in Figure 1. The intent was for the framework to be applicable to a wide variety of experimental systems, from pathogens to bioengineering to manipulation of the human body.

Because predicting when, in the future, any given theoretical capability of engineering biology may become a reality, the report developed a framework that focuses on determining what was possible at the that time and then deciphering what barriers and bottlenecks existed that, if overcome, would enable a new or emerging technology to be misused. It then applied the framework to pathogens, production of biologics or biochemicals, and alteration of the human host.

The result of this exercise was not a series of absolutes, but rather a relative ranking of what types of experimentation posed the highest vulnerabilities at the time.^21^ High on the list were recreating known pathogenic viruses, making existing viruses or bacteria more dangerous, producing chemicals *in situ* in microbes, and manufacturing chemicals or biological agents using known metabolic pathways. Lowest on the list was using gene drives to modify human populations.

The report highlighted several key challenges, including concerns about (a) malicious exploitation of cell-based manufacturing of industrial chemicals and pharmaceutical precursors; (b) creation or manipulation of pathogens; (c) creation of harmful microbiomes; and (d) directed targeting of individuals based on their genomic identity.

The report further recommended several actions that the U.S. government broadly, and the study's sponsor (DoD) more specifically, should consider.^21^ Among these recommendations are the following:

- “The Department of Defense and its partners in the chemical and biological defense enterprise should continue exploring strategies that are applicable to a wide range of chemical and biological threats.” The report stressed the need for the implementation of nimble strategies to keep pace with the rapid advances in technology research and development.
- The report recognized the challenges in predicting “how a synthetic biology-enabled weapon” could challenge existing monitoring and detection capabilities, and consequently requested that the DoD and its partners evaluate extant infrastructure to detect, identify, and notify natural and deliberate health threats.
- “The U.S. government, in conjunction with the scientific community, should consider strategies that manage emerging risk better than current agent-based lists and access control approaches.” The report recognized the limitations of existing agent-based policies in mitigating risks of new materials developed using engineering biology approaches.

The report also highlighted several areas to address challenges in assessing and mitigating potential risks from advances in synthetic biology. These areas include (a) development of means for detecting unusual patterns resulting from a “synthetic biology-enabled weapon”; (b) use of computational approaches for preventing, detecting, controlling, and attributing events involving organisms and/or materials developed using synthetic biology; and (c) use of synthetic biology (now referred to as engineering biology) to develop biodefense solutions, particularly to “advance detection, therapeutics, vaccines, and other medical countermeasures.”

Since this report was published, significant advances in engineering biology research and development have occurred and continue to be supported by governments, foundations, and the private sector. These investments contribute to economic initiatives focused on biotechnology and biomanufacturing, medicine, agriculture, and environmental sustainability.

Furthermore, research in related fields is pushing the boundaries of what is possible with engineering and more broadly, transdisciplinary, biology. The case studies, which focus on advances in biotechnology occurring after publication of the 2018 report, featured in the next section focus on research near these edges and explore the use of the framework presented earlier to keep pace with advances in the fields of microbiome, biohybrid materials, and generative AI.

## Case Studies: Scientific Advances in Engineering Biology and the Synthetic Biology Assessment Framework

### Microbiome Case Study

The original National Academies' framework considered alternations of human physiology, including through microbiomes, with a primary bottleneck considered to be a limited understanding of the microbiome itself. The areas the 2018 report described that would be necessary to overcome bottlenecks to use and/or misuse life sciences research included improvements in knowledge related to microbiome colonization of human hosts; *in situ* horizontal transfer of genetic elements; and a better understanding of host–microbe interactions or other relationships between microbiome organisms and host processes.

In the few short years since the report was released, many advances have overcome several of these bottlenecks. Microbiota, beyond fundamental support of digestion, have been found to exert profound effects on the immune and nervous systems, with implications for cognition, behavior, and vulnerability to infections. A recent National Academies' transdisciplinary workshop focused on the role of microbiota in the gut/brain axis revealed a rapid increase in understanding of the role of gut microbes, not only through computational and engineering models, but also through both animal and human studies of disease—including autism and rheumatoid arthritis.^9^

The FDA has already approved two microbiome treatments, and although they are focused on “balancing” the microbiome for digestive health,^32^ the number of potential therapeutic targets for the microbiome is significantly broad, and many early successful studies are progressing to Phase I/II clinical trials. In addition to a diversity of therapeutic targets, there is also a tremendous diversity of therapeutic approaches—including prebiotics, live biotherapeutics, bacteriophage cocktails, and fecal transplants, which can deliver different effective molecules or microbes.

These broad aspects could expand the “Usability of the Technology” according to the framework. However, given that most microbiome treatments are transient (2 weeks) at best, and require high consistent doses of microbe, the barrier to “Usability as a Weapon” could still remain.

### Biohybrid Materials

The convergence of material science and life sciences has shuttled in a new era of bioengineering to produce novel biohybrid materials and novel uses of existing biological materials using metals, ceramics, plastic, glass, and cells or tissues.^33^ Biological material is a general term that encompasses both engineered and synthetic material. Materials that are inspired by biological structures, characteristics, and processes and/or function, often referred to as bioinspired materials, do not necessarily have any biological components (e.g., living cells, biologically derived proteins, and nucleic acids).^34^

However, materials that are derived from or contain parts from living systems are referred to as biohybrid materials and can include a combination of nonliving materials and biological components.^35^ Biohybrid materials have several dynamic functions (i.e., structure, sense, responsivity, and reflectivity) that inspire their development for a variety of purposes such as medicine,^33,36–39^ textile,^8,40–42^ and construction.^43–48^

For example, the development of biological materials (e.g., cells) as inks to be used with three-dimensional (3D) printing equipment has enabled the creation of printed tissues and organs for regenerative medicine uses and/or laboratory-based experiments.^49,50^ More recently, research groups have adapted experimental 3D bioprinting to use both biological and non-biological components to create a variety of tissues, such as skin, skeletal muscle, brain tissue, bone, cartilage, and liver.^50,51^

Other studies involve the development of bioelectronic sensors and bioadhesives for monitoring physiological activity inside and on the skin of the human body; microbial fuel cells to power small devices; and dynamic biopolymers that may be responsive to their environments.^8^

Biohybrid materials can be developed through engineering biology or harnessing natural functions of microbes. Scientists predict that the Earth could contain 1 trillion microbial species or more.^52^ Only a very small number are known and only a fraction of these currently can be manipulated experimentally. In recent years, scientists have discovered and characterized microbes with unique functions, such as production of electricity, biomineralization, and carbon capture,^53–58^ and subsequently begun engineering biohybrid systems to harness a few of these functions for commercial and/or environmental sustainability purposes.

The products resulting from these efforts produce materials that have properties that traditional materials may lack. For example, organisms that produce composite materials with bio-produced fibers may be lower cost, lighter, and more ecologically friendly than materials developed using traditional petroleum-based development.^59^ Another example are products that use microalgae to harvest and store solar energy because of its higher efficiency at photosynthesis than plants.^55^

Although research identifying microbes with unique functions that may help to address societal challenges is very early stage, some companies have demonstrated initial success in harnessing these functions for product development, which has contributed to recent efforts toward green chemistry and the bio-based economy. In the United States, some of these companies were featured during the release of the 2022 *Executive Order on Advancing Biotechnology and Biomanufacturing Innovation for a Sustainable, Safe, and Secure American Bioeconomy*.

This Executive Order stresses the need to simultaneously promote safe and secure innovation in transdisciplinary biotechnology research and development to produce solutions for various challenges, such as “in health, climate change, food security, agriculture, supply chain resilience, and national and economic security,” and protect these innovations from theft and other unauthorized access and uses.^19^ Using the framework for assessing risks of synthetic biology,^21^ this article explores one aspect of this promote and protect paradigm—namely consideration about risk of malicious exploitation of two biohybrid materials, specifically engineered biohybrid materials and bioelectronics.

#### Building materials

Research on bioengineered living materials seeks to harness the unique functions of various different microbes. One such function is the ability of certain microbes to produce calcite precipitate through natural biochemical processes.^55^ Combining the microbes' natural biomineralization abilities with biochemical processes (i.e., urea hydrolysis, denitrification, and dissimilatory sulfate reduction), can produce strong and durable bioconcrete.

Some researchers are also developing these materials to create biocement, “self-healing” concrete, and bio-bricks. Other scientists are developing organisms that can produce bacterial cellulose that can sense and respond to stimuli and possibly self-repair for possible use in walls. Still other researchers are developing mycelium biocomposite materials as responsive and dynamic building materials.^8,55^

The risk of malicious exploitation of engineered biohybrid building materials likely is low because it still is in the early stages of research and development and has a high barrier to use, scientifically, technologically, from an infrastructure standpoint, and from the level of resources needed. Furthermore, exploitation of research and development in this field likely would not reflect traditional conceptions of biological weapons, specifically pathogens and biologically derived toxins.

However, these materials may provide well-resourced actors with options for building physical structures more rapidly than with conventional materials and for reducing the environmental footprint of traditional building materials. These uses likely do not present biological security concerns, although they may present broader national and economic security concerns if intellectual property, proprietary data and materials, and trade secrets are obtained in an illegal or unauthorized manner.

#### Bioelectronics

Bioelectronics encompasses a variety of research and development efforts including medical devices, which have been developed and commercialized for decades; bioadhesives that convert chemical signals inside the body to electronically readable signals outside the body (and sometimes through Wi-Fi); and electronic devices that incorporate biological materials to reduce reliance on external or battery power sources.^8,60^

Thus, the field of bioelectronics integrates living materials with electronics for various purposes and advances through transdisciplinary research and development. Although much of the research and development of bioelectronic devices appears to be in biomedicine, new research suggests that bioelectronics can be developed for various other fields beyond medicine.^8,61^ For example, recent research in biohybrid sensors and actuators integrate cells (e.g., bacterial, algal, and even mammalian cells), soft materials, and electronics for sensing and adapting to stimuli; harvesting, storing, and converting energy; and/or to enhance efficiency of miniaturized electronic systems.^8,62,63^

An example of recent research at the leading edge of bioelectronic research involves the use of living cells (specifically muscle cells) as actuators in a soft robot to power locomotion in response to a light signal.^63^ This soft robotic system balanced the engineering and physical properties with the active forces of muscle contraction and tension.^64,65^

Assessing the risks of malicious exploitation of bioelectronics is challenging, in part because of the breadth of research and technology development included in this broad field. Some advances, such as improvements to medical devices and wearable sensors may be low risk altogether in part because of the high level of scientific, technological, and resources needed to design and create them.

However, other advances, such as in soft robotics, may be associated with unanticipated risks, although none are similar to conventional biosecurity risks. As research and development in this field advances, critically thinking about the possible uses, identifying and prioritizing plausible risks, and taking steps to reduce those risks such that the benefits can be reaped may enable responsible innovation in these biohybrid systems.

### AI and Engineered Organisms

Although the concept of AI is nearly seven decades old, its rapid development and expansion in recent years has been a result of advances in neuroscience, specifically understanding how neural networks function, and the explosion of data generated through numerous sources. AI has evolved from sophisticated mathematical equations to billion-dollar investments in supercomputers that can play chess to computational algorithms that rely on training data.^66^

The emergence of ChatGPT and other generative AI models in 2022 has resulted in significant excitement about its potential to transform various sectors, including science, education, and health.^67^ Generative AI models are part of a broader category of foundation models and include LLM, which uses natural language processing and large amounts of data to generate text or other content. LLMs are a type of neural network that accesses and analyzes large amounts of text-based data to produce results in a manner that replicates human language.^68^

LLMs were first introduced to biology by DeepMind in 2021 when the company released AlphaFold, an LLM to predict protein structure, and holds promise in modeling other biological molecules and phenomenon. Scientists across a diversity of disciplines have begun applying LLMs and more specifically, generative AI models, to assist with everything from literature reviews to complex research, such as precision medicine.^69^

Despite these promises, the discourse about biosecurity risks—let alone legal risks (e.g., copyright infringement), privacy risks (e.g., access to personal data), ethical risks (e.g., amplification of bias inherent in the data),^70–74^ and scientific risks (e.g., generation of nonexistent or false information)^75^—has expanded significantly during the past year. Several organizations—Helena,^30^ Nuclear Threat Initiative,^29^ Federation of American Scientists,^28^ RAND Corporation,^27^ the InterAcademy Partnership,^76^ and the National Academies of Sciences, Engineering, and Medicine^26^—have published white papers, reports, and/or proceedings on biosecurity issues associated with generative AI in the life sciences.

Several of these reports highlight concerns about lowering barriers to designing and engineering organisms and a subset of these reports identify specific risks of accessing scientific methodologies, names of scientists with specific expertise, and genetic sequences that could aid in designing harmful pathogens. In addition, the National Academies' proceedings-of-a-workshop series in brief - highlights risks of using AI for drug discovery to design chemical weapons, which is described in a 2022 article^77^; privacy and security risks to individuals whose information (e.g., genetic and biometric information) is included in data sets; theft of data; and inaccurate or error-prone results from biased data and algorithms or manipulation of data sets.^26^

Concerns about the dual use nature of AI are not new, as scientists and security experts have described in recent years, even as early as 2014, when the American Association for the Advancement of Science, Federal Bureau of Investigation, and United Nations Interregional Crime and Justice Research Institute (AAAS-FBI-UNICRI) published a report that focused on national and transnational security risks and benefits associated with big data in the life sciences.^23^

This report considered benefits to various sectors, not only benefits to countering biological risks. Consideration about the implications of AI on biosecurity risks subsequently was raised by the United Nations Institute for Disarmament Research in its 2020 report on biosecurity implications of advances in science and technology^24^ and by IEEE in its 2021 article on responsible innovation of AI in the life sciences and biotechnology research enterprise.^25^

Largely driven by questions about risk, most existing frameworks for assessing “dual use” issues focus on the risk that peaceful research could be exploited by a malicious actor to cause harm, but rarely on the benefits of the research in addressing existing and future biological risks. Frameworks initially developed after the publication of the 2004 National Academies report on *Biotechnology Research in an Age of Terrorism* focused on research activities that could provide the information (e.g., function of specific genes in different organismal backgrounds, mutations and their effects, or methodologies) that may be of interest to malicious actors interested in using biology to cause harm.^22^

Frameworks developed to assess risks and benefits of “gain-of-function” research nearly a decade later focused on quantitatively assessing the risk of accidental exposure of respiratory pathogens, semi-quantitatively assessing the risk of deliberate exposure or release of pathogens, and qualitatively assessing the risk of exploiting information published in the scientific literature and of the benefits of research involving pathogens.^78^

Shortly thereafter, the National Academies' 2018 report *Biodefense in an Age of Synthetic Biology* describes a conceptual framework for assessing biosecurity risks of synthetic biology, which focused on characterizing the ability of technologies to be used by malicious actors, the ability for the technology or product to be used as a weapon itself, the ability of the malicious actors to use the technologies; and the existence of approaches for risk mitigation.^21^ In 2020, biosecurity experts described an adaptation to a framework produced from the previously described AAAS-FBI-UNICRI effort to enable risk assessments from convergence of scientific disciplines with the life sciences (i.e., bio + X).^79^

These analyses and frameworks vary significantly in the types of risks and consequences assessed, and their ability to assess and balance both risk and benefit of a technology or scientific advance, all of which influence the discourse about security risks associated with exploitation of the use of AI in the life sciences and biotechnology. Furthermore, they are not always informed by true experts in computer science, specifically AI algorithms and/or biological concepts, systems, data, and data sets.

These domain experts provide a solid understanding of the limitations and capabilities of AI models (perhaps still not individual algorithms), the underlying data and knowledge used by the models (either for training and validation or for analysis), and the veracity of the final analytic research, which often is difficult to confirm using independent methods.

In its 2023 *Executive Order on the Safe, Secure, and Trustworthy Development and Use of Artificial Intelligence*, the White House requested a study by the National Academies to: “(A) assesses the ways in which AI can increase biosecurity risks, including risks from generative AI models trained on biological data, and makes recommendations on how to mitigate these risks; (B) considers the national security implications of the use of data and data sets, especially those associated with pathogens and omics studies, that the United States Government hosts, generates, funds the creation of, or otherwise owns, for the training of generative AI models, and make recommendations on how to mitigate the risks related to the use of these data and data sets; (C) assesses the ways in which AI applied to biology can be used to reduce biosecurity risks, including recommendations on opportunities to coordinate data and high-performance computing resources; and (D) considers additional concerns and opportunities at the intersection of AI and synthetic biology that the Secretary of Defense deems appropriate.”^31^

This requested study explicitly focuses on the multiuse nature of AI by requesting an assessment of its biosecurity risks and capabilities for reducing biosecurity risks.

## Conclusion

The field of engineering biology continues to advance at a pace that challenges current risk assessment frameworks. Most extant biological security frameworks, including the Synthetic Biology Assessment Framework, focus on risks of using advanced approaches and technologies to enhance or create novel pathogens and toxins. At their core, these frameworks focus only on risk and include elements consistent with identifying, assessing, and mitigating risks.

The Synthetic Biology Assessment Framework differs from previous risk assessment frameworks by its inclusion of capability considerations, specifically the ability for an actor to use the technology, the ability of the technology to create and deliver a weapon, the expertise and resources of actors, and sufficiency of policies and programs to prevent, deter, mitigate, and/or attribute an event caused by the use of the technology to create and/or deliver a weapon.

These elements are consistent with generalized risk assessment methodologies that equate risk to probability and consequence of development and dissemination. With the lens of conventional biological agents, this framework likely is sufficient to assess new science and technology advances. However, if the technologies are not usable with conventional biological agents and the risks are to economic or broader national security, perhaps even this risk framework is limited in its applicability.

Furthermore, none of these frameworks examine benefits of the technology, either to countering chemical, biological, radiological, and/or nuclear risks or to addressing critical societal, scientific, and environmental challenges, and currently, few, if any, frameworks evaluate risks and benefits together, largely because a vast majority of the discourse has been focused primarily on risk. Although in addition to developing a risk-based framework, the National Academies' report on *Biodefense in an Age of Synthetic Biology* recommended the development of engineering biology to provide innovative solutions for biological threats.

These considerations are of particular relevance as the U.S. Executive Order on biotechnology and biomanufacturing, Bold Goals for biomanufacturing, and the Engineering Biology Research and Development initiative in the CHIPS and Science Act seek to advance research and development in engineering biology fields that address various critical societal challenges.

## Authors' Contributions

All authors contributed equally to the development and writing of this article.
